# S08-3: Goals and realities in local governments’ involvement in PA promotion for diverse populations subgroups – a comparative analysis of municipalities from 5 countries

**DOI:** 10.1093/eurpub/ckae114.234

**Published:** 2024-09-26

**Authors:** Petru Sandu, Peter Gelius, Sven Messing, Antoine Noel-Racine, Yuko Oguma, Tanja Onatsu, Antonia Paula Papiu, Yoshinobu Saito, Noriko Takeda, Katariina Tuunanen, Anne Vuillemin, Razvan Mircea Chereches

**Affiliations:** Friedrich-Alexander-Universität Erlangen-Nürnberg, Department of Sport Science and Sport, Erlange, Germany; Institut des Sciences du Sport, Université de Lausanne; Friedrich-Alexander-Universität Erlangen-Nürnberg, Department of Sport Science and Sport, Erlange, Germany; Physical Activity for Health research group, Health Research Institute, Physical Education and Sport Sciences department, Ireland; LAHMESS, Université Côte d‘Azur; Keio University; JAMK University of Applied Sciences; University Babes-Bolyai, Department of Public Health; Keio University; Kogakuin University; JAMK University of Applied Sciences; LAHMESS, Université Côte d‘Azur; University Babes-Bolyai, Department of Public Health

## Abstract

Although cross-sectoral cooperation is important at all levels, in practice it often remains at a declarative level. Different sectoral policies and the resulting measures to promote HEPA are usually disconnected, uncoordinated and without the necessary synergies. This pertains especially to local environments with less financial and expert support, such as small rural communities, where people have few HEPA options that are suitable and attractive for vulnerable groups: children and adolescents, elderly and people with disabilities.

“Development of indicators for the assessment of the provision of settlements with green spaces for outdoor physical activity” is a research project co-funded by the Slovenian Research and Innovation Agency, the Ministry of Health and the Ministry of Natural Resources and Spatial Planning and implemented at the Urban Planning Institute of the Republic of Slovenia. The aim of the project is to define indicator(s) for the provision of green spaces for PA and active lifestyles in settlements, which could be used at local level to assess, improve, and monitor the situation. We took a medium-sized city and a small rural community as pilot projects, the latter often being a disadvantaged environment in terms of HEPA in spatial planning compared to larger cities. On the pilots, our objectives have been to establish cooperation at the local level between at least three different sectors and to test methodology and indicators developed in the project to assess the provision of green spaces to promote PA in selected environments, thus enabling more equitable and inclusive spatial conditions for HEPA at the local level.

The presentation will present the approach, results and overall experiences with the work implemented in the pilot project of a rural community. It will highlight the benefits of cross-sectoral cooperation for more effective local level HEPA policies to ensure the inclusive and equitable access to green spaces in settlements.

**Funding source:**

Slovenian Research and Innovation Agency, grant no. V5-2232

